# Accelerating Hyperparameter Tuning in Machine Learning for Alzheimer’s Disease With High Performance Computing

**DOI:** 10.3389/frai.2021.798962

**Published:** 2021-12-08

**Authors:** Fan Zhang, Melissa Petersen, Leigh Johnson, James Hall, Sid E. O’Bryant

**Affiliations:** ^1^ Institute for Translational Research, University of North Texas Health Science Center, Fort Worth, TX, United States; ^2^ Department of Family Medicine, University of North Texas Health Science Center, Fort Worth, TX, United States; ^3^ Department of Pharmacology and Neuroscience, University of North Texas Health Science Center, Fort Worth, TX, United States

**Keywords:** machine learning, hyperparameter tuning, alzheimer’s disease, high performance computing, support vector machine

## Abstract

Driven by massive datasets that comprise biomarkers from both blood and magnetic resonance imaging (MRI), the need for advanced learning algorithms and accelerator architectures, such as GPUs and FPGAs has increased. Machine learning (ML) methods have delivered remarkable prediction for the early diagnosis of Alzheimer’s disease (AD). Although ML has improved accuracy of AD prediction, the requirement for the complexity of algorithms in ML increases, for example, hyperparameters tuning, which in turn, increases its computational complexity. Thus, accelerating high performance ML for AD is an important research challenge facing these fields. This work reports a multicore high performance support vector machine (SVM) hyperparameter tuning workflow with 100 times repeated 5-fold cross-validation for speeding up ML for AD. For demonstration and evaluation purposes, the high performance hyperparameter tuning model was applied to public MRI data for AD and included demographic factors such as age, sex and education. Results showed that computational efficiency increased by 96%, which helped to shed light on future diagnostic AD biomarker applications. The high performance hyperparameter tuning model can also be applied to other ML algorithms such as random forest, logistic regression, xgboost, etc.

## Introduction

Alzheimer’s disease (AD) is the most common form of dementia. In 2020, as many as 5.8 million Americans were living with AD. This number is projected to nearly triple by 2060 (Prevention, 2021). Machine Learning (ML) methods for AD and AD Related Dementias (ADRDs) is growing faster than ever before (Waring et al., 2008; Magnin et al., 2009; O'Bryant et al., 2011a; O'Bryant et al., 2011b; O'Bryant et al., 2013; O'Bryant et al., 2014; Weiner et al., 2015; O'Bryant et al., 2016; O'Bryant et al., 2017; Grassi et al., 2018; Hampel et al., 2018; O'Bryant et al., 2018; Stamate et al., 2019; Zetterberg and Burnham, 2019; Zhang and Sejdić, 2019; Franzmeier et al., 2020; O'Bryant et al., 2020; Rodriguez et al., 2021). A PubMed search using keywords of AD and ML showed that the number of publications related to ML for AD has increased by 146 percent from just two in 2006 to 294 in 2020. For example, O'Bryant et al. developed a Support Vector Machine (SVM) model with 398 plasma samples obtained from adults with Down syndrome to predict incident mild cognitive impairment (MCI) (AUC = 0.92) and incident AD (AUC = 0.88) (O'Bryant et al., 2020). O’Bryant et al. also developed a precision medicine model for targeted NSAID therapy in AD based on data collected from a previously conducted clinical trial. This work included 351 patients with mild-to-moderate AD that were enrolled into one of three trial arms: 1-year exposure to rofecoxib (25 mg once daily), naproxen (220 mg twice-daily) and placebo. The SVM model yielded 98% theragnostic accuracy in the rofecoxib arm and 97% accuracy in the naproxen arm, respectively (O'Bryant et al., 2018). Magnin et al. also built a SVM model with three-dimensional T1-weighted MR images of 16 patients with AD and 22 elderly controls and obtained a 94.5% mean accuracy for AD with a mean specificity of 96.6% and mean sensitivity of 91.5% (Magnin et al., 2009).

Improving speed and capability is a huge issue in applying ML to AD. It is possible that certain ML computations can be delayed because of the large amount of time to iteration that is required, for example, time to train with hyperparameters tuning. High performance computing (HPC) can be used to help meet the increasing demands for the speed and capabilities of processing ML for AD (Eddelbuettel, 2021). With the fast processing ability of high-performance computing systems, faster results can be delivered, which in turn would not only speed up finding the optimal hyperparameters for AD with ML models but would also identify opportunities to fix issues in hyperparameter tuning for AD ML models.

In this paper, based on the multicores parallel structure of Talon3 high performance computing provided by the University of North Texas, we present a high performance computing workflow to support our parallel SVM hyperparameter tuning. We applied the multicore high performance SVM hyperparameter tuning to 100 times repeated 5-fold cross-validation model for longitudinal MRI data of 150 subjects with 64 subjects classified as demented and 86 subjects classified as nondemented. The computational time was dramatically reduced by up to 96% for the high performance SVM hyperparameter tuning model. The multicores parallel structure and the high performance SVM hyperparameter tuning model can be used for other ML applications.

## Materials and Methods

### Parallel Structure

We used the Talon3 system (Table 1) provided by University of North Texas for this study due to its convenient computing services, which allowed us to import/export/execute large and complex parallel ML. The hardware configuration of the Talon3 contains the following: more than 8,300 CPU cores, 150,000 GPU cores, Mellanox FDR InfiniBand network, and over 1.4 Petabytes of Lustre File Storage. The amount of AD data necessary for performing ML with a PC workstation is massive. For example, in one study with 300 samples, to process just the 100 times repeated 5-fold cross-validation for hyperparameters tuning with SVM, it would require about 3 h of consecutive CPU time and 12 GB of storage with a local computer.

**TABLE 1:** Talon3 computer nodes.

Quanity	Memory (GB)	Cores	Description
192	64	28	Dell PowerEdge C6320 server with two 2.4 GHz Intel Xeon E5-2680 v4 14-core processors
75	32	16	Dell PowerEdge R420 server with two 2.1 GHz Intel Xeon E5-2450 eight-core processors
64	64	16	Dell PowerEdge R420 server with two 2.1 GHz Intel Xeon E5-2450 eight-core processors
8	512	32	Dell PowerEdge R720 server with four 2.4 GHz Intel Xeon E5-4640 eight-core processors
16	64	28	Dell PowerEdge R730 server with two 2.4 GHz Intel Xeon E5-2680 v4 14-core processors and two Nvidia Tesla K80 GPUS (4,992 GPU cores/card)

For parallel computing, the Talon3 provides several options including: SNOW, Rmpi, and multicore. We chose multicore because it executes parallel tasks on a single node as opposed to multiple nodes and the level of flexibility is higher than the other two options. For multicore parallel programming, submitting high performance ML includes two parts: a shell script and an R script. The shell script we submitted for multicore is for a single node with 28 cores in C6320. And for the R script high performance ML, we used doParallel (Michelle Wallig et al., 2020; Eddelbuettel, 2021) and foreach (Michelle Wallig and Steve, 2020; Eddelbuettel, 2021) packages.

### Parallel SVM Hyperparameter Tuning

**FIGURE 1:**
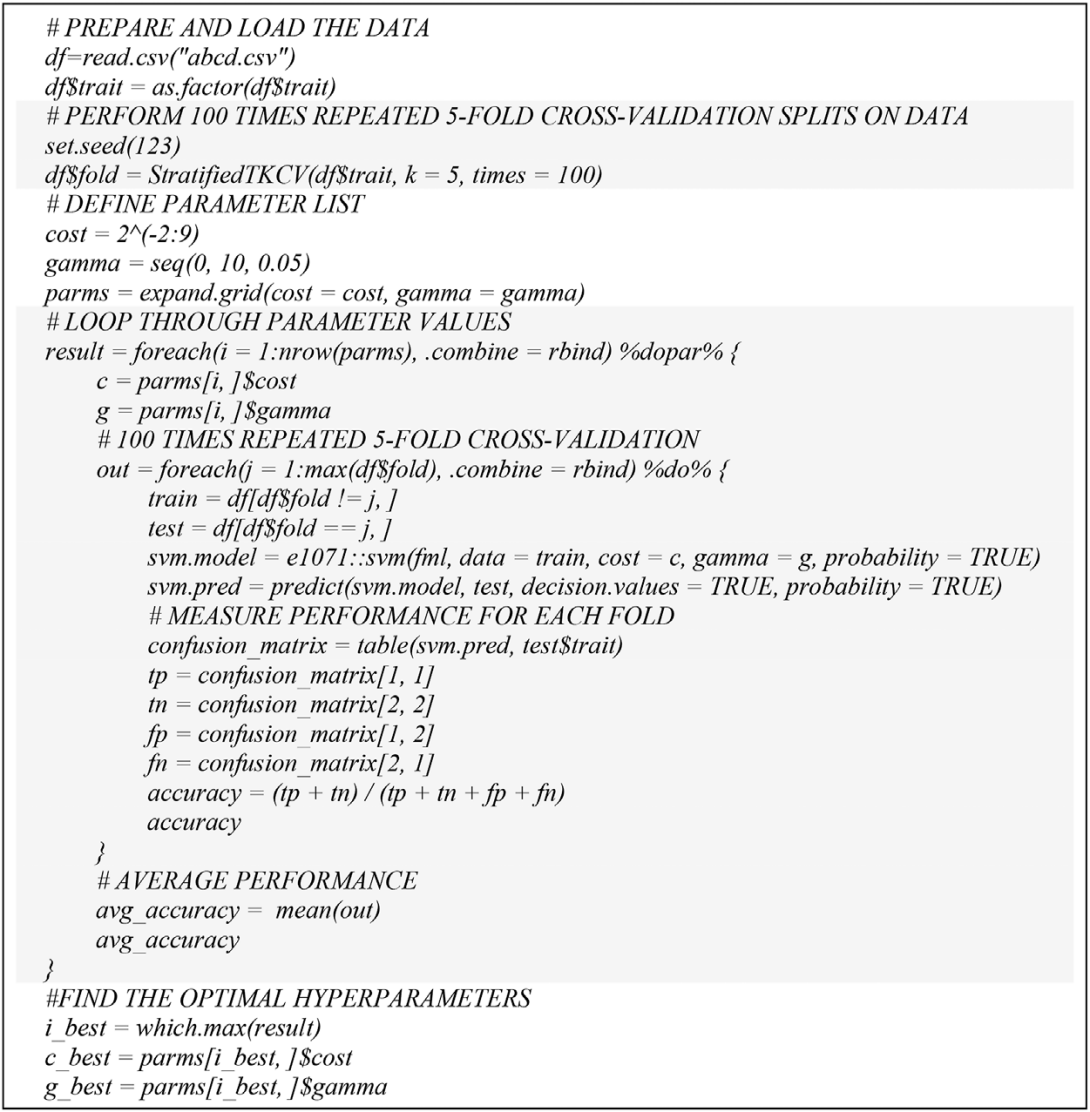
Pseudo code for parallel SVM hyperparameter tuning.

Based on the above parallel structure in Talon3 and doParallel package, we developed a high performance computing workflow to support our parallel SVM hyperparameter tuning (Figure 1). We used a grid search approach to find the best model parameters in terms of accuracy. This procedure mainly contains three steps: 1) define a grid to vary cost and gamma, 2) perform 100 times repeated 5-fold cross-validation splits on training data, and 3) tune the cost and gamma of the SVM model.

### 100 Times Repeated 5-Fold Cross-Validation

A single run of the 5-fold cross-validation (O'Bryant et al., 2019) may result in a noisy estimate of model parameters. We adopted 100 times repeated 5-fold cross-validation (Kublanov et al., 2017) to improve the estimation of optimal parameters of the ML model. This involves simply repeating the cross-validation procedure 100 times and reporting the mean performance across all folds from all runs. This mean performance is then used for the determination of optimal parameters.

### Metrics

The following eight measurements were involved in our evaluation: 1) Sensitivity (also called recall), the proportion of actual positive pairs that are correctly identified; 2) Specificity, the proportion of negative pairs that are correctly identified; 3) Precision, the probability of correct positive prediction; 4) Accuracy, the proportion of correctly predicted pairs; 5) Area Under the Curve; 6) Negative Predictive Value (NPV), the probability that subjects with a negative screening test truly don’t have the disease; 7) Negative Predictive Value at base rate of 12% (NPV12); and 8) Positive Predictive Value at base rate of 12% (PPV12).

## Results

We downloaded open access longitudinal MRI data available on nondemented and demented older adults (Marcus et al., 2010a). The dataset consisted of longitudinal MRI data from 150 subjects aged 60 to 96. 72 of the subjects were classified as “nondemented” throughout the study. 64 of the subjects were classified as “demented” at the initial visit and remained so throughout the study. 14 subjects were classified as “nondemented” at the initial visit and were subsequently characterized as “demented”at a later study visit. For each subject, three to four individual T1-weighted magnetization prepared rapid gradient-echo (MP-RAGE) images were acquired in a single imaging session (Marcus et al., 2010b). The subject-independent model we developed for parallel hyperparameter tuning is not based on a classifier trained for each subject individually. We chose the following five imaging and clinical variables to predict the status of AD: SES (Socioeconomic Status), MMSE (Mini Mental State Examination), eTIV (Estimated Total Intracranial Volume), nWBV (Normalize Whole Brain Volume), and ASF (Atlas Scaling Factor). Measurements of these variables in this cohort including clinical dementia rating scale (CDR), nWBV, eTIV, ASF, etc. have been previously described elsewhere (Marcus et al., 2010b). Three demographic variables: Sex, Age, and Edu (Years of education) were also added as covariates.

**FIGURE 2:**
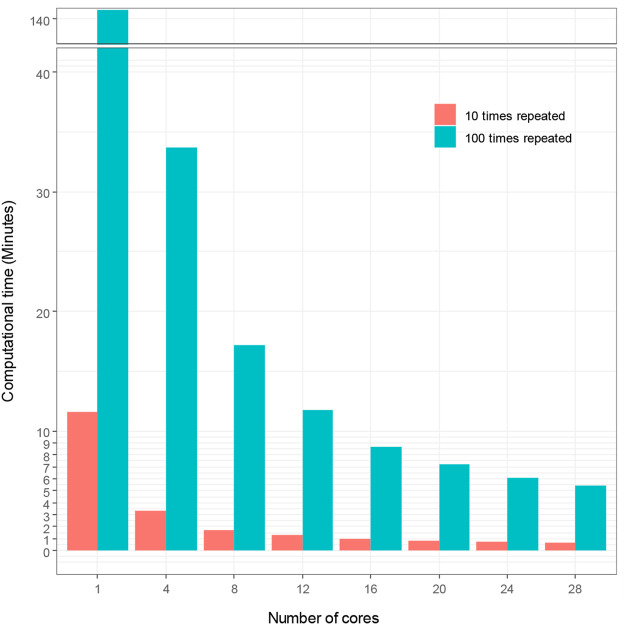
Computational time vs. number of cores with SVM modeling.

We used sbatch commands to submit shell scripts and R scripts for comparing computational time for hyperparameter tuning under different number of cores and repeated times of 5-fold cross-validation in Talon3. With SVM modeling, we demonstrate in Figure 2 how the number of cores affects the computational time of hyperparameter tuning, which shows that the computational time decreases proportionally as the number of cores increases. Figure 2 also demonstrates that the repeated times of the 5-fold cross-validation algorithm for hyperparameter affects the computational time. The computational time increased proportionally with the increasing repeated times. The time spent initially for the hyperparameter tuning without using high performance computing is very large (140.73 min for *t* = 100; 11.58 min for *t* = 10). The computational time decreased to 5.44 and 0.67 min, for *t* = 100 and for *t* = 10 respectively, when we used 28 cores to accelerate hyperparameter tuning in ML. We thereby reduced computational time by up to 96%, with high performance ML model.

The optimal hyperparameters we obtained are the same for all runs (gamma = 0.005, cost = 32). We used grid search method and set boundary for the two parameters: cost and gamma as suggested in the paper (Hsu et al., 2003) where fine grid search was on cost = (2, 32) and gamma = [2^(−7), 2^(−3)]. We extended the cost boundary to (0.25, 512) and the gamma boundary to (0, 10) to catch as much change as possible. Variables importance under the SVM model with the optimal hyperparameters shows that the MMSE, nWBV, and SES are leading variables in predicting dementia (AD) status. Out of the three demographic variables, education was shown to be less important for the SVM model than Age and Sex.

With the optimal hyperparameters, the average performance that the SVM model achieved for a testing set of 12 Demented and 17 Nondemented is reported on below for both 100 times repeated 5 -fold cross-validation and 10 times repeated 5 -fold cross-validation. The performance (Table 2) is slightly higher than previously reported at https://www.kaggle.com/hyunseokc/detecting-early-alzheimer-s, which achieved accuracy = 0.82, sensitivity = 0.70, and AUC = 0.82 for SVM. Our results show that the high performance SVM hyperparameter tuning workflow that we presented can significantly reduce computational time while maintaining the necessary accuracy.

**TABLE 2:** Performance for testing set after hyperparameter tuning.

	Actual demented	Actual nondemented
Predicted demented	9	1
Predicted nondemented	3	16
Precision/PPV	90.00%	
Accuracy	86.21%	
Sensitivity	75.00%	
Specificity	94.12%	
NPV	84.21%	
AUC	90.80%	
PPV12	63.49%	
NPV12	96.50%	

**FIGURE 3:**
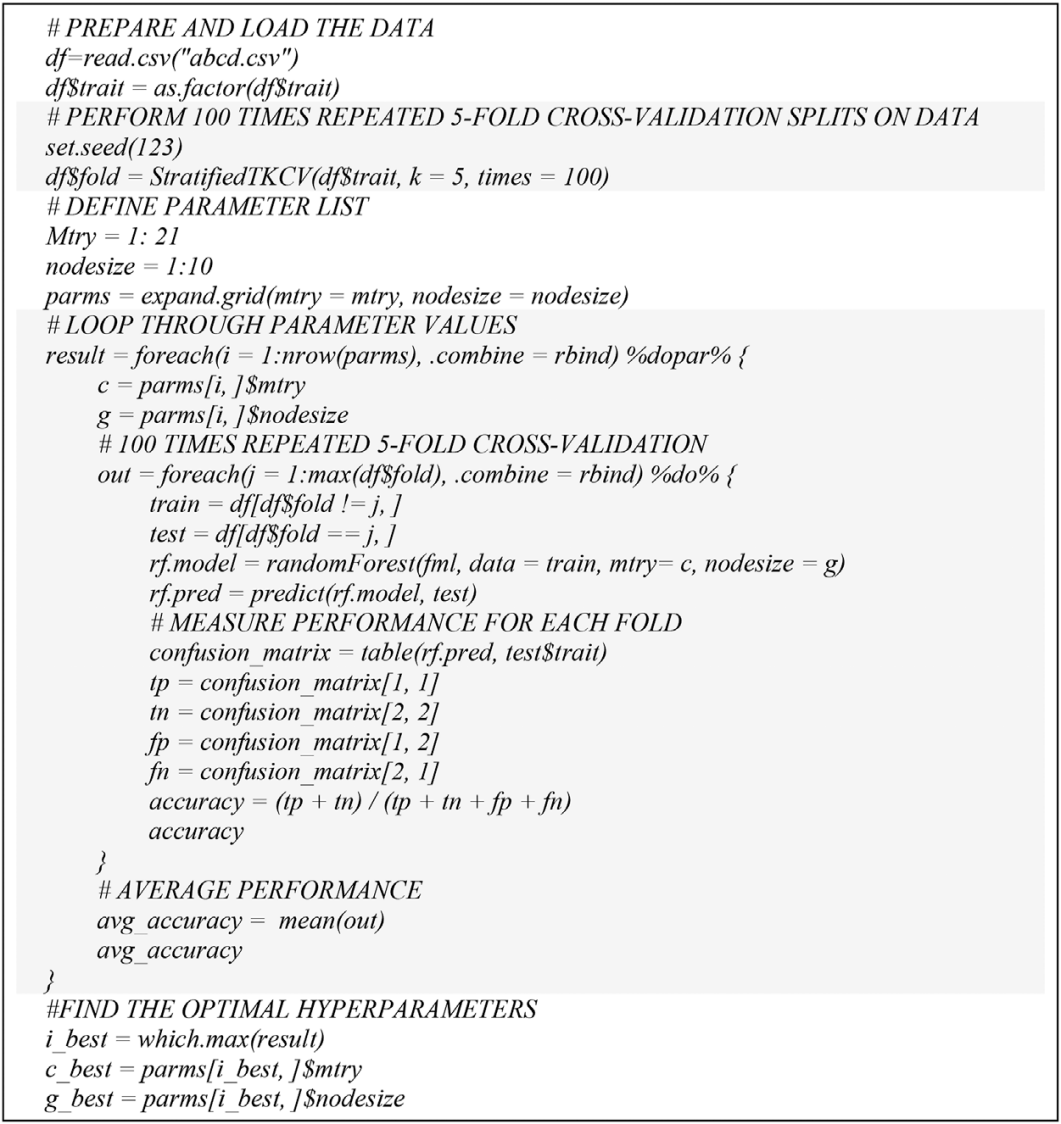
Pseudo code for parallel RF hyperparameter tuning.

**FIGURE 4:**
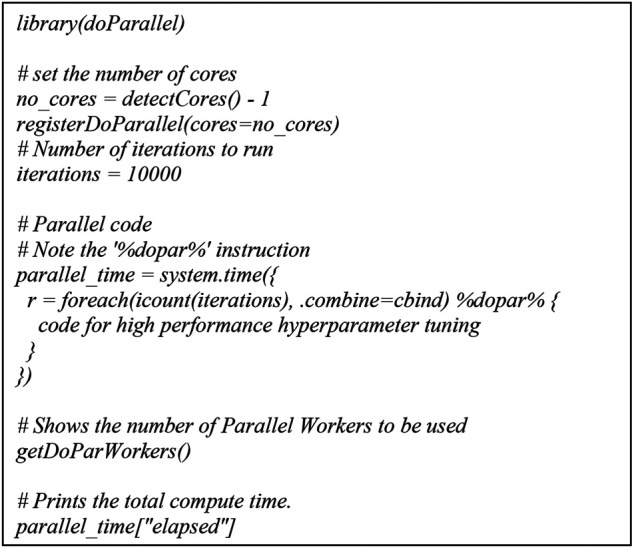
R script for parallel computing.

**FIGURE 5:**
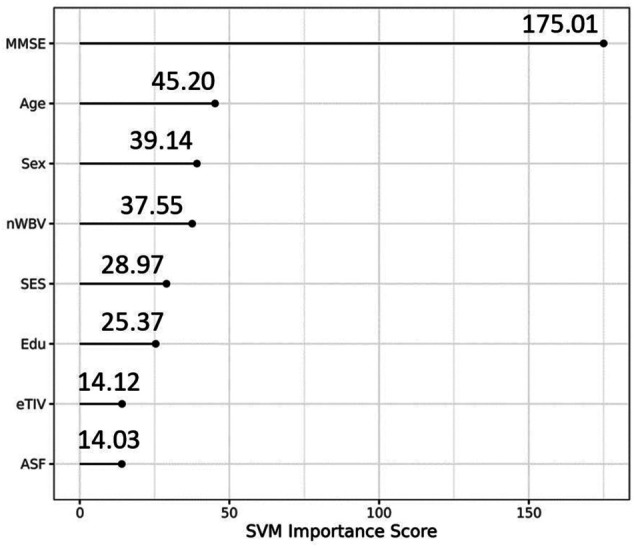
Variable importance of the eight variables.

In order to demonstrate the extensibility of our hyperparameter tuning workflow to other ML models, we also followed the SVM hyperparameter tuning workflow (Figure 3) and adopted random forest into our parallel hyperparameter tuning workflow (Figure 4). We obtained consistent results that the computation time for hyperparameter tuning of random forest was also remarkedly reduced (Figure 5). The computational time was reduced from 47.67 to 2.24 min by 95% and from 5.25 min to 18.17 s by 94%, for *t* = 100 and *t* = 10 respectively.

We also tested the adaptability of our hyperparameter tuning workflow to the Texas Alzheimer’s Research and Care Consortium (TARCC) dataset (Zhang et al., 2021). The TARCC dataset contains a total of 300 cases (150 AD cases; 150 Normal Control cases). Each subject (at one of the five participating TARCC sites) undergoes an annual standardized assessment, which includes a medical evaluation, neuropsychological testing, and a blood draw. The same blood-based biomarkers in (Zhang et al., 2021) were used as features for parallel hyperparameter tuning. Even when adopting a new TARCC dataset into our parallel hyperparameter tuning workflow (Figure 1), we obtained consistent results, which showed that the computation time for the hyperparameter tuning of the new TARCC dataset was also remarkedly reduced by about 96%. The computational time was reduced from 311.5 to 12.48 h by 96% and from 34.03 to 1.6 h by 95%, for *t* = 100 and *t* = 10 respectively.

## Discussions

HPC advances have successfully helped scientists and researchers to achieve various breakthrough innovations in the field of Omics-medicine, technology, retail, banking and so on (Merelli et al., 2014). For example, HPC has been applied to Next Generation Sequencing that is extremely data-intensive and needs ultra-powerful workstations to process the ever-growing data (Schmidt and Hildebrandt, 2017). Hyperparameter tuning component of ML can be a high-performance computing problem as it requires a large amount of computation and data motion. ML requires a computationally-intensive grid search and lots of computational power to help enable faster tuning cycles. Introducing HPC to ML can take advantage of high volumes of data as well as speed up the process of hyperparameter tuning.

Therefore, we presented a parallel hyperparameter tuning workflow with HPC to exploit modern parallel infrastructures to execute large-scale calculations by simultaneously using multiple compute resources. The rationales are 1) the foreach package that the workflow is based on supports parallel execution and provides a new looping construct for executing R code repeatedly. Specifically, a problem is broken into discrete parts that can be solved concurrently and an overall control/coordination mechanism is employed; 2) the foreach package can be used with a variety of different parallel computing systems, include NetWorkSpaces and snow; and 3) foreach can be used with iterators, which allows the data to be specified in a very flexible way.

**FIGURE 6:**
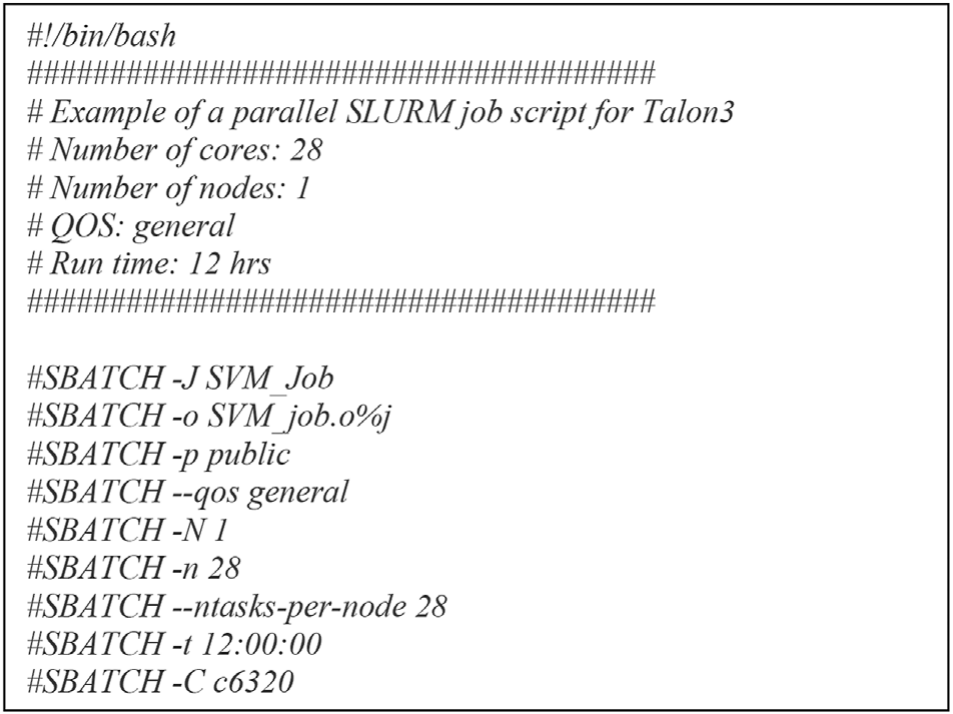
Shell script for parallel computing.

The multicore high performance SVM hyperparameter tuning workflow we presented is hardware-agnostic and can be used in HPCs of most U.S. universities or commerical clouds for example, Amazon AWS, Microsoft Azure, Google Cloud, etc. Before executing the multicores high performance SVM hyperparameter tuning, R package (V4.0.3, Linux) and doParallel and foreach libraries should be installed successfully, which are met for HPCs in most U.S. universities or commerical clouds. There are mainly two of the most popular job schedulers used for requesting resources allocations on a multi user cluster: 1) the Simple Linux Utility for Resource Management (Slurm) and 2) the Portable Batch System (PBS). In Figure 6, we described the shell script for parallel computing for the Slurm system. Similary a shell script for parallel computing for PBS system is as followed.

The multicore high performance SVM hyperparameter tuning workflow significantly reduced computational time while maintaining a consistent detection accuracy. The workflow was diagrammed through a multicore computing pseudo code using the doParallel package in R for high performance hyperparameter tuning. The basic idea of multicore computing is to allow a single program, in this case R, to run multiple threads simultaneously in order to reduce the “walltime” required for completion. The doParallel package in R is one of several “parallel backends” for the foreach. It establishes communication between multiple cores, even on different physical “nodes” linked by network connections. The foreach function evaluates an expression for each value of the counter (iterator) “case”. The %dopar% operator is used to execute the code in parallel. Using %do% instead would lead to sequential computation by the primary process. When parallelizing nesting for loops, there is always a question of which loop to parallelize. If the task and number of iterations vary in size, then it’s really hard to know which loop to parallelize. We parallelized the outer loop in our SVM hyperparameter tuning because this would result in larger individual tasks, and larger tasks can often be performed more efficiently than smaller tasks. The hyperparameter tuning could be parallelized at the inner loop also if the outer loop doesn’t have many iterations and the tasks are already large.

**FIGURE 7:**
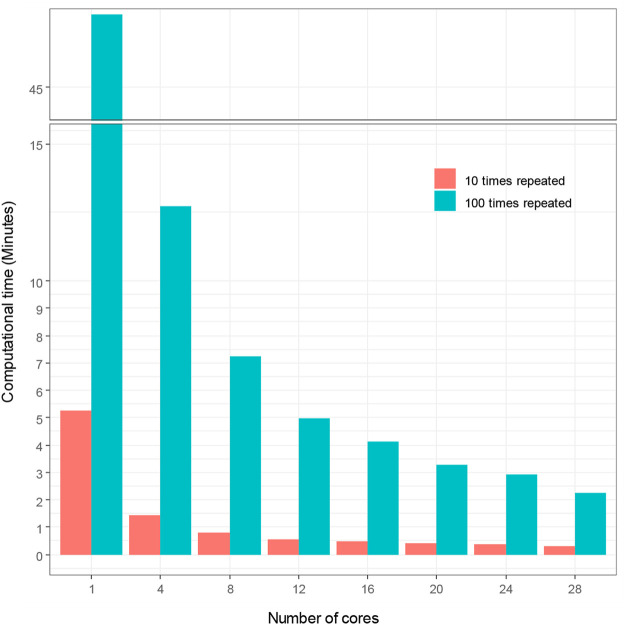
Computational time vs. number of cores with RF modeling.

The multicores high performance hyperparameter tuning workflow can also be used for other ML such as random forest, logistic regression, xgboost, etc. For example, we demonstrated that a random forest model can be adopted into our parallel hyperparameter tuning model (Figure 3) and the results we obtained were consistent in that the computation time for hyperparameter tuning of random forest models were remarkedly reduced (Figure 7). In the future, we plan to use Rmpi library to create multinodes parallel computing workflow for hyperparameter tuning when Talon3 supports multinodes parallel computing to run R script.

Our optimal hyperparameter model also showed that MMSE, Age, Sex, nWBV, and SES are important variables in AD diagnosis, which is consistent with previous findings. For example, Arevalo-Rodriguez et al. found that baseline MMSE scores can achieve a sensitivity of 76% and specificity of 94% for predicting conversion from MCI to dementia (in general) and a sensitivity of 89% and specificity of 90% for predicting conversion from MCI to AD dementia (Arevalo-Rodriguez et al., 2015). Advanced age and sex are two of the most prominent risk factors for dementia. Females are more likely to be susceptible for developing AD dementia than males (Podcasy and Epperson, 2016). Podcasy at Penn PROMOTES Research on Sex in Health and examined sex and gender differences in the development of dementia and suggested that researchers should consider sex as a biological variable for dementia research (Podcasy and Epperson, 2016). Rose et al. evaluated the combination of cerebrospinal fluid biomarkers with education and normalized whole-brain volume (nWBV) to predict incident cognitive impairment (Roe et al., 2011). They concluded that time to incident of cognitive impairment is moderated by education and nWBV for individuals with normal cognition had higher levels of cerebrospinal fluid tau and ptau at baseline (Roe et al., 2011). Khan et al. and Leong et al. assessed the role of various features on the prognosis of AD, and found that sex, age, MMSE, nWBV, and SES were significantly associated with and made an impact on the occurrence of AD (Leong and Abdullah, 2019; Khan and Zubair, 2020).

## Data Availability

Publicly available datasets were analyzed in this study. This data can be found here: https://www.oasis-brains.org.
